# Cord Blood Adductomic Profiling Provides Preliminary Insights on Perinatal Smoking Exposure: A Pilot Analysis

**DOI:** 10.3390/antiox15091078

**Published:** 2026-08-28

**Authors:** Janu Newar, Elizabeth Brees, Erika T. Lin, Abhik Chakraborty, Aliyah Abanes, Kathleen M. Fisch, William Funk, Karen K. Mestan

**Affiliations:** 1 Department of Pediatrics, Division of Neonatology, University of California San Diego, 9500 Gilman Drive, La Jolla, CA 92093, USA; jnewar@health.ucsd.edu (J.N.); ebrees@health.ucsd.edu (E.B.); a4chakraborty@health.ucsd.edu (A.C.); a1abanes@health.ucsd.edu (A.A.); 2 Department of Obstetrics/Gynecology and Reproductive Sciences, University of California San Diego, 9500 Gilman Drive, La Jolla, CA 92093, USA; kfisch@health.ucsd.edu; 3 Department of Preventive Medicine, Northwestern University, 680 North Lake Shore Drive, Suite 1400, Chicago, IL 60611, USA; w-funk@northwestern.edu

**Keywords:** maternal smoking, perinatal oxidant stress, preterm birth, neonatal outcome

## Abstract

Maternal smoking during pregnancy (MSDP) remains a significant public health concern. The molecular mechanisms underlying smoking-induced maternal-fetal impact remain incompletely understood. Newer omics platforms, such as cord blood adductomics, can provide preliminary insight into the “perinatal exposome” leading up to birth. The objective of this study is to explore the adductomic signatures of oxidant stress in cord blood associated with MSDP. In a cohort of mother-infant dyads enrolled at a single birth center in Chicago, IL (2008–2021), we conducted a secondary analysis of self-reported maternal smoking exposure data that was linked to an existent adductomics database of 105 addition products (adducts) measured in cord blood plasma. Principal component analysis (PCA), volcano plots and PERMANOVA were used to visualize the variability in adductomic profiles and to compare significantly increased and decreased adduct concentrations according to self-reported current-smokers, former-smokers, and never-smokers. N = 158 participants (N = 6 current-smokers, 26 former-smokers, and 126 never-smokers) had documented self-reported smoking status and were included in the analysis. PCA demonstrated separation between current-smokers and the other groups (R^2^ = 0.026 for current vs. never-smokers, R^2^ = 0.115 for current vs. former-smokers; Padj = 0.0075 for both) and no difference between former vs. never-smokers. Among 56 annotated adducts, 27 were increased in current vs. never-smokers, and 32 in current vs. former-smokers. Mode of delivery (*p* = 0.02) and maternal hypertension (*p* = 0.001) were significantly different among the three groups. Matched analysis by chronic hypertension (yes/no) supported the main finding that current-smoker adductomic profiles were distinct from both former (*p* = 0.015) and never-smoker (*p* = 0.0225) profiles. Self-reported MSDP appears to be associated with cord blood adductomic signatures of oxidant stress. Further studies are needed to determine how the duration, extent and timing of exposure correlate with exposomic changes at birth.

## 1. Introduction

Maternal smoking during pregnancy (MSDP) represents a preventable cause of adverse perinatal outcomes, including fetal growth restriction, preterm birth, stillbirth and placental complications [1,2]. Despite declining smoking rates in many developed countries, approximately 7–10% of pregnant women continue to smoke, with higher rates in certain demographic groups [3,4]. Cord blood, representing the offspring circulation at the time of birth, provides a unique opportunity to explore the potential impact of maternal-fetal exposures to environmental toxicants such as tobacco smoke [5].

While the epidemiological associations between smoking and adverse pregnancy outcomes are well established [2,6], the molecular mechanisms underlying smoking-induced dysfunction remain incompletely characterized. Adductomic profiling is an emerging approach for identifying biochemical signatures that may elucidate mechanisms of environmental exposures [7,8,9,10,11,12,13]. Specifically, adductomics measures the formation of multiple chemical addition products (adducts), which are covalent modifications of biomolecules by reactive compounds. These adductomic profiles represent a complex molecular fingerprint of oxidative stress exposure [13,14].

We previously described cord blood adductomic profiles associated with preterm birth and neonatal outcome, focusing on bronchopulmonary dysplasia [15]. A marginally significant (*p* = 0.05) but unexpected association between preterm birth and maternal smoking status was identified in that cohort, which prompted us to explore environmental exposures such as MSDP in the next step. Thus, the objective of the current study was to perform a secondary analysis to better understand how adductomics profiles differ in a subsample of the cohort for which maternal smoking status may potentially play a role in perinatal oxidative stress.

## 2. Materials and Methods

### 2.1. Patient Population and Study Sample

The patient population was drawn from a larger cohort of mother-infant dyads prospectively enrolled at Prentice Women’s Hospital in Chicago, IL, USA. The database used at the time of this analysis included mothers and infants enrolled from 2008 to 2021, for which both maternal and infant outcomes data were completely extracted from electronic medical records. The inclusion/exclusion criteria, enrollment and data collection procedures for the original birth cohort have been previously published [15]. All study subjects provided informed consent prior to participation, and the study was approved by the Institutional Review Board of Northwestern University (protocol number STU00201858).

Of the complete adductomics dataset of 256 samples, 210 were cord blood specimens; the remaining 46 were peripheral blood samples. Selection of the 210 births was based upon the criteria and specific infant outcomes of the original funded prospective study (R21HD100831), in which cord blood from 79 preterm infants with BPD, 80 without BPD, and 51 healthy full-term infants were prospectively enrolled over the 2-year funding period.

**Figure 1:**
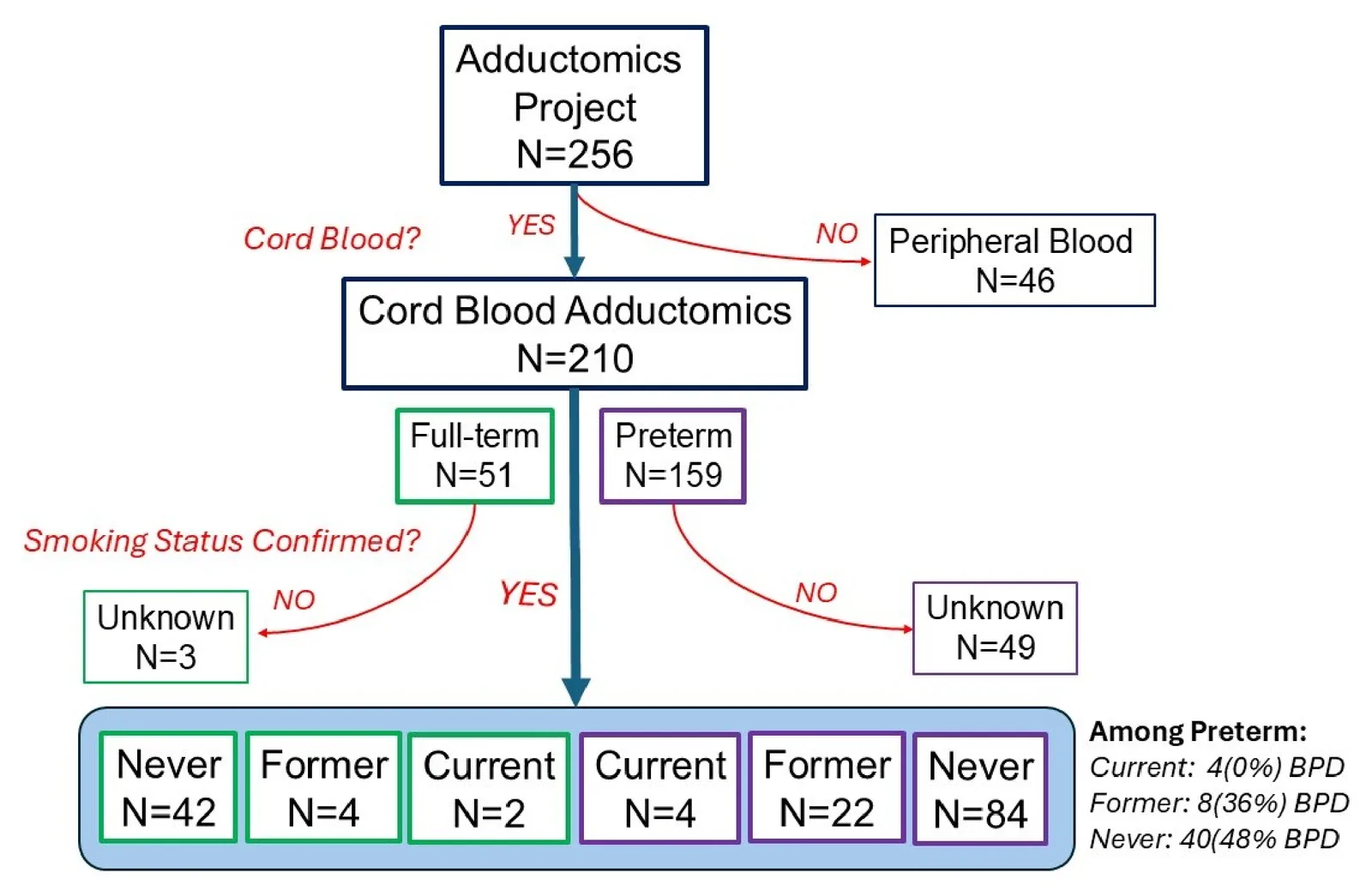
Decision tree demonstrating the criteria and selection process for the N = 158 participants included in the secondary analysis for MSDP. From the original 256 births with complete adductomics data, 210 had cord blood adductomics completed. Smoking status was confirmed in 48 (94%) of full-term births and 110 (69%) of the preterm births (which were all extremely preterm, born at ≤28 completed weeks gestational age). Among the preterm births, 0% of current-smokers, 36% of former-smokers and 48% of never-smokers gave birth to infants who developed BPD. Green indicates full-term births and purple indicates preterm births; blue shading indicate the cord blood samples and smoking-status subgroups retained in the final analytic cohort.

The study sample for the current analysis included the subset of participants from the above adductomics database with known maternal smoking status as determined by self-report at the time of admission to the labor and delivery unit (below). Of the 210 births with cord blood adductomic data, 158 had documented maternal smoking status and were included in the present analysis (48 of 51 full-term births: 42 never, 4 former, and 2 current smokers; and 110 of 159 preterm births: 84 never, 22 former, and 4 current smokers). The remaining 52 participants (3 full-term, 49 preterm) lacked documented smoking status and were excluded. A breakdown of the cohort and inclusion/exclusion criteria for the study sample selected for the secondary analysis are shown in Figure 1.

### 2.2. Determination of Maternal Smoking Status

In the current study, we linked maternal smoking status data extracted from the social history section of the electronic medical records. Standardized data were obtained via self-report at the time of admission to the Labor and Delivery Unit from the patient intake form in EPIC, and included the following fields: (1) Never Smoker; (2) Former Smoker; (3) Current Every Day Smoker; (4) Current Some Day Smoker; (5) Never Assessed; (6) Unknown if Ever Smoked. Maternal and infant clinical data were linked to the cord blood adductomics data via unique study codes without use of personal identifiers. Included in the present study were those births in which complete maternal smoking data were entered (i.e., complete self-reported entry in all relevant fields of the admission note). Maternal smoking status was categorized as “current smoker” (included current every day and someday smoking status), “former smoker” (quit prior to pregnancy or at least >30 days prior to admission), or “never smoker.” These data, along with all clinical data below, were extracted from electronic medical records using automated protocols in consultation with Northwestern’s Enterprise Data Warehouse (EDW). The study was approved by the institutional review board, and all participants provided informed consent.

### 2.3. Clinical Data Collection

Maternal and neonatal characteristics were abstracted from electronic medical records, including gestational age at delivery, birth weight, birth weight percentile, infant sex and neonatal outcomes. Maternal preeclampsia and chorioamnionitis were defined according to American College of Obstetrics and Gynecology (ACOG) criteria [16,17].

### 2.4. Sample Collection and Processing

Cord blood samples were collected immediately following delivery, processed and archived per an established protocol with human serum albumin (HSA) isolated from plasma as previously published [15]. This included a standardized protocol of plasma separation (via refrigerated table-top centrifuge, 3000 rpm × 10 min) from EDTA tubes, aliquoting and immediate storage at −80 °C until the assay [18]. The entire adductomics dataset of 256 blood samples was completed in a single run, which minimized batch effects. The isolated HSA was subjected to trypsin digestion (porcine pancreas, lyophilized powder; Sigma-Aldrich, St. Louis, MO, USA) following established methodologies for adductomic analysis [15].

### 2.5. Adductomic Analysis

Untargeted discovery of unknown adducts was performed using high-resolution mass spectrometry (HRMS) (Thermo Fisher, San Jose, CA, USA), and targeted analyses of known adducts were performed using highly sensitive triple quadrupole mass spectrometry (QQQ-MS) (Agilent Technologies, Santa Clara, CA, USA). A total of 56 known (annotated) adducts were quantified, including various cysteine modifications, oxidation products, and conjugates. Both adductomic approaches focused on HSA-Cys^34^ adducts, which serve as sentinel biomarkers of electrophilic exposure [15,19]. Unknown adducts were identified based on mass-to-charge ratio (*m*/*z*) and retention time using untargeted adductomic methods [19] (see Supplementary Data S1).

### 2.6. Statistical Analysis

Parametric and nonparametric tests were used to compare continuous data, where appropriate. Categorical data were compared using Fisher’s exact or Chi-square. Adduct concentrations are reported in pmol of adduct/mg HSA, which were converted from adduct peak areas/HKP peak area (PAR) values using two external calibration curves. (see Lin et al., [15] Supplementary Figure S1). All adduct concentration data were non-normally distributed and log10-transformed to approximate normal distribution where appropriate. In contrast to our previous publication, no normalization to a reference denominator was applied prior to transformation. For principal component analysis (PCA), the log10-transformed adduct concentrations were mean-centered and unit-variance scaled, using the prcomp function in R (Version 4.5.2), to visualize overall adductomics patterns across smoking groups. The R code used for data analysis has been provided in Supplementary Data S2.

PERMANOVA (Permutational Multivariate Analysis of Variance) (adonis2 function, R package vegan v2.7.5) was used to test for differences in adductomic profiles between groups, based on Euclidean distances calculated from the log10-transformed adduct concentration matrix [20]. PERMDISP (betadisper function, R package vegan v2.7.5) was used to assess homogeneity of multivariate dispersion between groups, based on the same distance matrices [21]. This test evaluates whether within-group variability (distance to group centroid) differs between groups, which can otherwise confound interpretation of PERMANOVA results as reflecting differences in group centroids. An omnibus three-group test (never vs. former vs. current-smoker) was performed prior to the three pairwise comparisons (current vs. never-smokers, current vs. former-smokers, and former vs. never-smokers). All PERMANOVA models used 999 permutations with a fixed random seed (20260804) to ensure reproducibility. Pairwise PERMANOVA *p*-values were adjusted for multiple comparisons using the Benjamini-Hochberg (BH) procedure. R^2^ values were calculated to determine the proportion of variance explained by smoking status.

For individual adduct analysis, an omnibus Kruskal–Wallis test was used to compare median concentrations across the three smoking-status groups; pairwise comparisons of individual adduct concentrations between groups (current vs. never, current vs. former, and former vs. never) were subsequently performed using two-sided Wilcoxon rank-sum (Mann–Whitney) tests, which generated the *p*-values shown in the volcano plots below. Fold-change for each adduct was calculated as the difference in group medians on the log10 scale, converted to log2 scale (log2FC = log10-scale median difference/log10(2) [22]. Ninety-five percent confidence intervals for log2 fold change estimates were obtained via nonparametric bootstrap resampling (1000 replicates, resampling with replacement within each group, 2.5th–97.5th percentile method) (Supplementary Data S3). Volcano plots were generated showing the relationship between fold-change (Log2 scale) and statistical significance (−Log10 adjusted *p*-value) [23]. Significance thresholds were set at adjusted *p*-value < 0.05 (corresponding to −Log10 *p* = 1.3) and |Log2 fold change| >1.0; adducts were classified as significantly differentially abundant only if they met both criteria. Multiple testing correction was performed using the Benjamini-Hochberg false discovery rate method, applied separately within each of the three pairwise comparisons and separately for known and unknown adducts [24]. For adducts meeting significance criteria, violin plots (with overlaid boxplots and individual sample points) were generated showing the distribution of adduct concentration (log10-scaled axis) by smoking status for the relevant pairwise comparison. For adducts significant in more than one pairwise comparison, the relationship between fold-change magnitudes across comparisons was assessed using Pearson correlation. Comparative analyses were performed for three pairwise comparisons: current vs. never-smokers, current vs. former-smokers, and former vs. never-smokers. Statistical significance was set at *p* < 0.05.

Known adducts were annotated with descriptive names via a lookup table cross-referenced by adduct identifier. Unknown adducts are reported as unannotated candidate signals, identified by mass shift and retention time (see Supplementary Data S1). All statistical analyses were performed in R (Version 4.5.2) using the packages vegan (v2.7.5), readxl (v1.4.5), ggplot2 (v4.0.2), ggrepel (v0.9.7), gridExtra (v2.3), dplyr (v1.2.0), tidyr (v1.3.2), and writexl (v1.5.4). The analysis code used is provided as Supplementary Data S2.

## 3. Results

### 3.1. Characteristics of the Patient Sample

Linkage of smoking status data with the cord blood adductomics database resulted in identification of 158 births with complete MSDP data available. (see Figure 1 below) Thus, the current analysis included 6 (3.8%) current smokers, 26 (16.5%) former smokers, and 126 (79.7%) never smokers. Maternal and infant characteristics by smoking exposure status are presented in Table 1. Gestational age and birth weight were similar across groups, with a predominance of preterm (mean gestational age: 30.9 ± 5.9 weeks) and low birth weight (mean birth weight: 1745.5 ± 1166.6 g) deliveries reflecting the high-risk population. Overall, maternal and infant characteristics did not differ significantly by smoking status, except for maternal chronic hypertension (*p* = 0.001) and mode of delivery (C-section vs. vaginal birth; *p* = 0.02).

### 3.2. Overall Adductomics Patterns by Smoking Status

**Figure 2:**
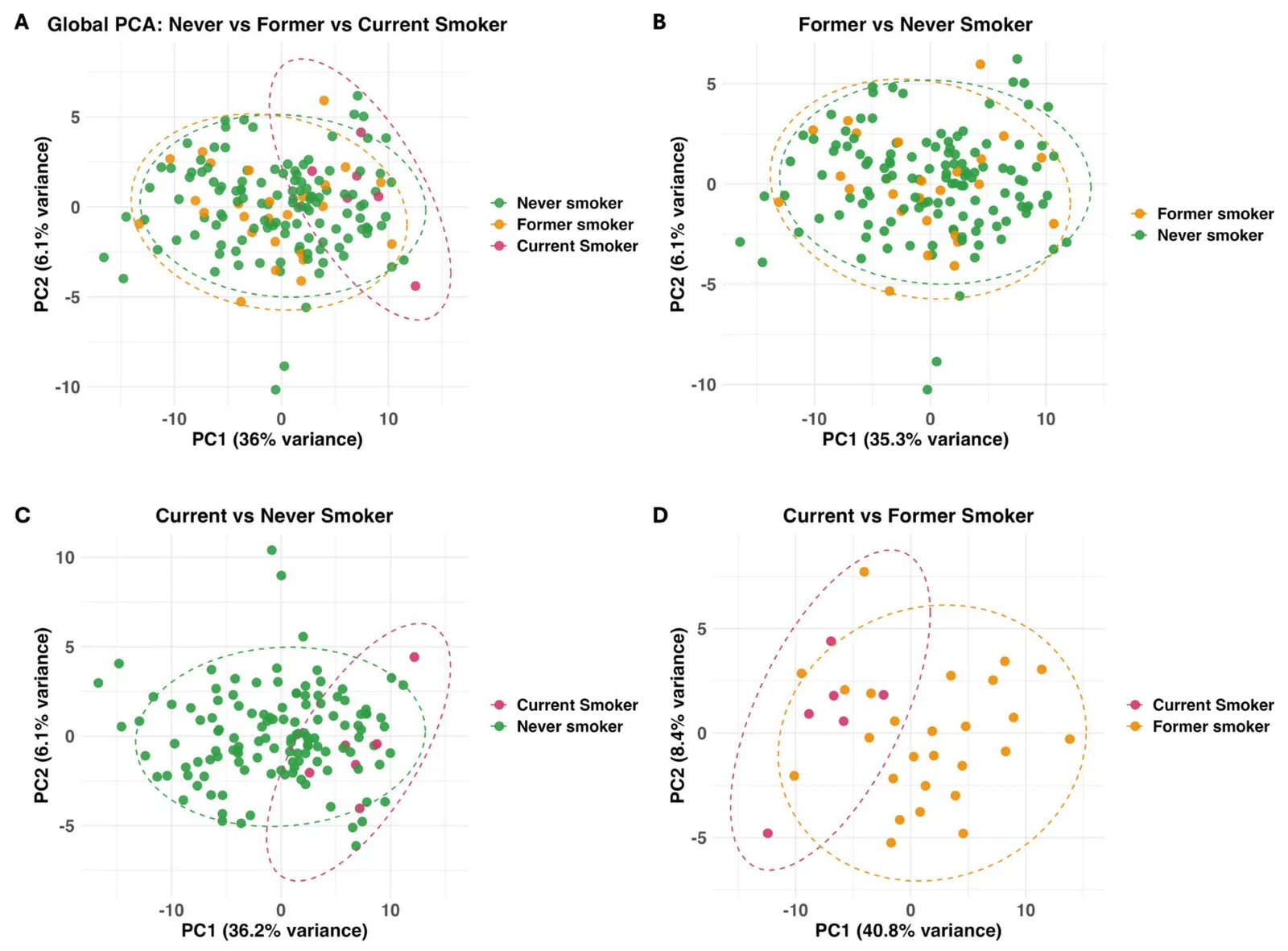
**Principal component analysis of cord blood adductomic profiles by maternal smoking status.** PCA plots showing separation of samples based on adductomic signatures. Each point represents an individual birth colored by smoking exposure group: Current-smoker (red), former smoker (yellow), never-smoker (green). (**A**) Global analysis demonstrating all 3 MSDP categories. (**B**) PCA of former vs. never-smokers. (**C**) PCA of current vs. never-smokers. (**D**) PCA of current vs. former-smokers.

PCA revealed differences in multivariate profile patterns based on maternal smoking status (Figure 2). An omnibus three-group PERMANOVA indicated differences in adductomic profiles by smoking status (R^2^ = 0.027, *p* = 0.009; Figure 2A and Table 2). Multivariate dispersion did not differ significantly between groups for the omnibus comparison or any pairwise comparison (PERMDISP: overall *p* = 0.149; never vs. former *p* = 0.152; never vs. current *p* = 0.138; former vs. current *p* = 0.522; Table 2), indicating no statistically significant evidence of differences in multivariate dispersion was detected between groups. PERMANOVA, with BH correction applied across the three comparisons, demonstrated no significant difference between former and never-smokers (Figure 2B; R^2^ = 0.004; adjusted *p* = 0.882), but distinct differences between current- versus never-smokers (Figure 2C; R^2^ = 0.026; adjusted *p* = 0.0075) and current vs. former-smokers (Figure 2D; R^2^ = 0.115; adjusted *p* = 0.0075).

### 3.3. Differential Annotated (Known) Adduct Formation

**Figure 3:**
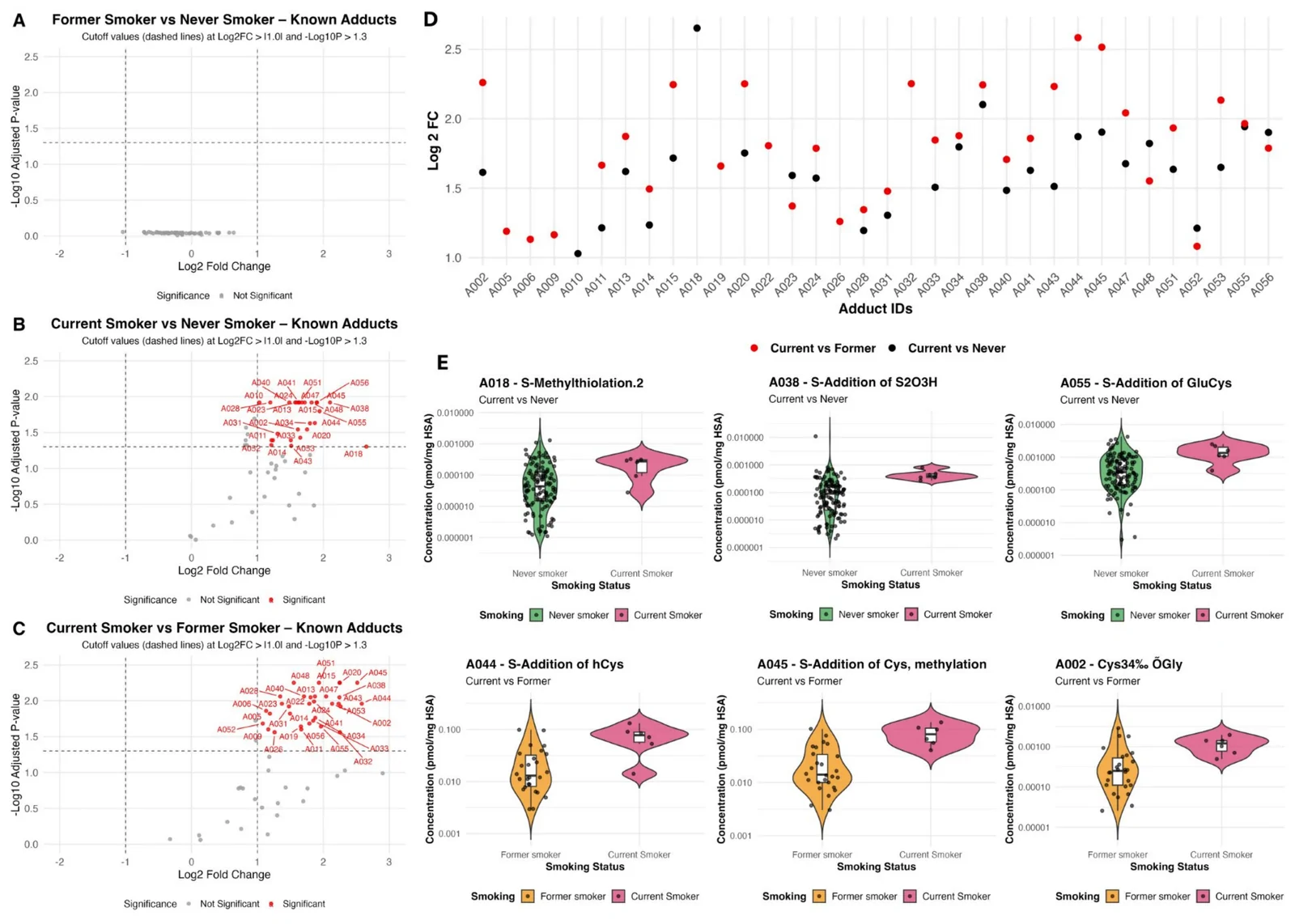
**Differential known adduct abundance and comparative fold-change analysis across MSDP groups.** Volcano plots display the relationship between fold-change (x-axis, Log2 scale) and statistical significance (y-axis, −Log10 adjusted *p*-value) for known adducts differentially abundant between groups. (**A**) Former vs. never-smokers. (**B**) Current vs. never-smokers, revealing 27 significantly increased adducts (red). (**C**) Current vs. former-smokers, showing 32 significantly increased adducts (red). Horizontal dashed line indicates adjusted *p*-value threshold of 0.05 (−Log10 *p* = 1.3), and vertical dashed lines mark Log2 fold-change thresholds of ±1.0. Points are colored by significance: gray (not significant) and red (significant). (**D**) Scatter plot comparing Log2 fold-changes for individual adducts significant in either comparison, with black circles showing current vs. never-smokers and red circles showing current vs. former fold-changes. (**E**) Violin plots illustrating concentration distributions of six representative adducts: top row shows current vs. never-smokers; bottom row shows current vs. former-smokers. Never-smokers shown in green, former-smokers shown in orange, and current-smokers shown in red.

Volcano plot analysis identified several adducts that appear altered in current-smokers compared to both other groups (Figure 3A–C). No significant adducts were identified in the former vs. never-smoker comparison, consistent with PCA findings. The current vs. never-smoker comparison revealed 27 significantly increased adducts, with no downregulated adducts. Two adducts were unique to this comparison: A010 (S-sodiation) and A018 (S-methylthiolation.2). The current vs. former-smoker comparison showed 32 significantly increased adducts, also with no downregulated adducts (Supplementary Data S3A). Seven adducts were unique in this comparison: A005 (T3 dimer), A006 (unmodified T3), A009 (methylation, not at Cys^34^), A019 (S–(O)–O–CH3), A022 (Cys^34^ sulfonic acid trioxidation), A026 (methylisocyanate adduct), and A032 (S-addition of mercaptoacetic acid). Twenty-five adducts were significantly altered in both comparisons.

Figure 3D displays the comparative fold-changes across both comparisons, with Log2FC values ranging from approximately 1.03 to 2.65 for current vs. never-smokers and 1.08 to 2.58 for current vs. former-smokers. The high degree of overlap between the two comparisons, with the majority of adducts showing similar directional changes, underscores the consistent adductomic signature of current smoking, with 25 of 34 altered adducts (74%) significant in both comparisons and fold-change magnitudes positively correlated between comparisons (r = 0.67). Key adducts with the highest fold-changes included A056 (S-addition of GSH) with Log2FC = 1.90 for current vs. never and Log2FC = 1.79 for current vs. former, A053 (Na adduct of S-CysGly) with Log2FC = 1.65 for current vs. never and Log2FC = 2.13 for current vs. former, and A048 (S-hCys, plus methylation not Cys^34^) with Log2FC = 1.82 for current vs. never-smokers and Log2FC = 1.55 for current vs. former-smokers. Other adducts with notable increases included A045 (S-addition of Cys, methylation) with Log2FC = 1.90 for current vs. never-smokers and Log2FC = 2.52 for current vs. former-smokers, A022 (Cys^34^ sulfonic acid trioxidation) which was uniquely significant in current vs. former-smokers with Log2FC = 1.81, and A028 (S-addition of crotonaldehyde) with Log2FC = 1.20 for current vs. never-smokers and Log2FC = 1.35 for current vs. former-smokers. Violin plots demonstrating these adduct distributions are shown in Figure 3E.

### 3.4. Unknown Adduct Discovery

**Figure 4:**
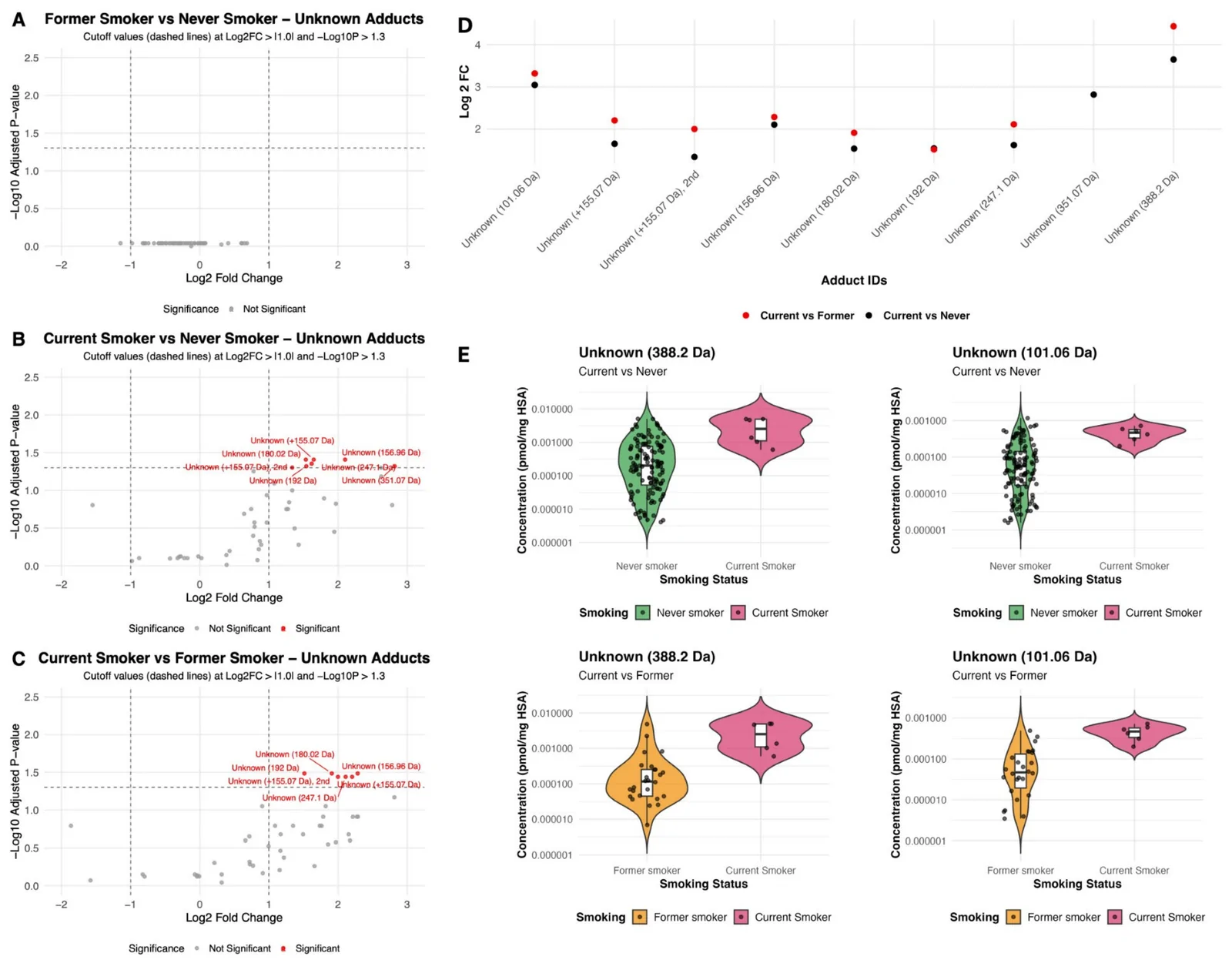
**Differential unknown adduct abundance and comparative fold-change analysis across MSDP groups.** Volcano plots display the relationship between fold-change (x-axis, Log2 scale) and statistical significance (y-axis, −Log10 adjusted *p*-value) for unknown adducts differentially abundant between groups. (**A**) Former vs. never-smokers, showing no significant unknown adducts. (**B**) Current vs. never-smokers, revealing 9 increased unknown adducts (red), including a unique feature at 351.07 Da. (**C**) Current vs. former-smokers, showing 8 increased adducts (red). Horizontal dashed line indicates adjusted *p*-value threshold of 0.05 (−Log10 *p* = 1.3), and vertical dashed lines mark Log2 fold-change thresholds of ±1.0. Points are colored by significance: gray (not significant) and red (significant). (**D**) Scatter plot comparing Log2 fold-changes for individual unknown adducts significant in either comparison, with black circles showing current vs. never and red circles showing current vs. former fold-changes. (**E**) Violin plots illustrate concentration distributions of the two unknown adducts with the largest fold-changes. Never-smokers are shown in green, former-smokers are shown in orange, and current-smokers are shown in red.

Analysis of unknown adducts identified additional MSDP-related adducts (Figure 4A). For current vs. never-smokers (Figure 4B), 9 unknown adducts were increased, with one unique feature at 351.07 Da. For current vs. former-smokers (Figure 4C), 8 unknown adducts were increased (Supplementary Data S3B). The fold-change patterns for unknown adducts revealed *m*/*z* features ranging from 101.06 Da to 388.2 Da. Unknown (351.07 Da) was unique in the current vs. never-smoker comparison, while Unknown (388.2 Da) showed the highest fold-change (Log2FC = 3.65 for current vs. never, Log2FC = 4.44 for current vs. former) (Figure 4D). Of the 9 altered unknown adducts, 8 were significant in both comparisons, all showing the same direction of change, with high fold-change magnitudes correlated between comparisons (r = 0.963). The identification of unknown adducts highlights the potential for discovering novel biomarkers of tobacco toxicant exposure.

### 3.5. Consideration of Potential Confounders

To evaluate the extent to which the observed adductomic differences by MSDP might be influenced by chronic hypertension and mode of delivery, we performed a matched case-control analysis, manually matching the 6 current-smokers 1:1:1 to 6 former-smokers and 6 never-smokers, based on maternal hypertension (yes/no). Where feasible, subjects were also matched by gestational age (mean difference 0.24–0.55 weeks across comparisons) and infant sex with one exception, yielding a balanced analytic subset of 18 patients (6 per group).

**Figure 5:**
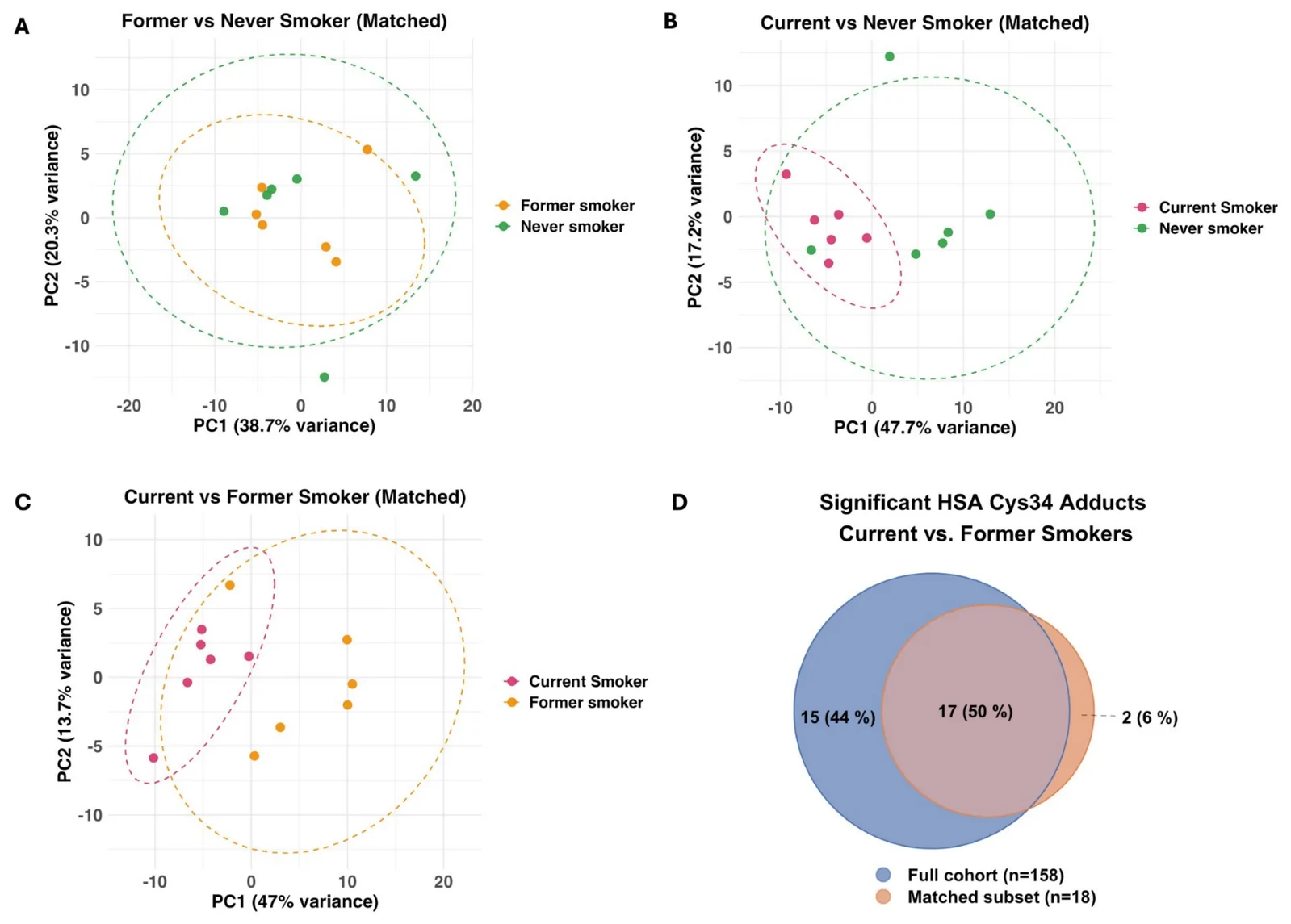
**Matched case-control analysis.** Subgroup analysis of a subset of 18 births (6 current-smokers: 6 former-smokers: 6 never-smokers) matched by maternal chronic hypertension (yes/no). (**A**–**C**): Pairwise PCA plot comparisons. (**D**): Venn diagram showing overlap of known adducts in the full (N = 158) and matched (N = 18) analysis.

PCA showed group separation patterns consistent with the full study sample (N = 158), with current-smokers showing separation from both former- and never-smokers along PC1 (current vs. never: PC1 = 47.7% variance; current vs. former: PC1 = 47.0% variance), while former and never-smokers showed overlap (former vs. never: PC1 = 38.7% variance) (Figure 5A–C). An omnibus 3-group PERMANOVA confirmed significant differences in profiles in the matched cohort (*p* = 0.014), driven by significant pairwise separation for current vs. never-smokers (adjusted *p* = 0.0225) and current vs. former-smokers (adjusted *p* = 0.015), with no significant difference between former- and never-smokers (*p* = 0.943). Multivariate dispersion (PERMDISP) did not differ significantly between groups in the matched cohort for any comparison (*p* > 0.45), indicating that the centroid separation was not attributable to unequal within-group variability.

At the individual-adduct level, 19 known adducts were significantly increased in current vs. former-smokers within the matched sample, compared with 32 in the full (unmatched) sample; 17 (50%) of these adducts overlapped between both analyses, while 15 were unique in the larger unmatched sample and 2 in the matched subset (Figure 5D). No known adducts were significantly different for current vs. never-smokers or former vs. never-smokers in the matched sample.

We also performed univariate analysis of mode of delivery (C-section vs. vaginal) in both the matched subset and the full sample. Delivery mode was not significantly associated with overall adductomic profiles in either cohort (PERMANOVA: N = 18, *p* = 0.660; N = 158, *p* = 0.133).

## 4. Discussion

We conducted a secondary analysis of a unique adductomics dataset to explore exposure biomarkers measured at birth that may be associated with MSDP. The adducts included in this analysis are implicated in pathways of oxidative stress based upon their distinct biochemical structures, as previously published [15]. In total, all 105 adducts were re-analyzed from the previous publication, with certain adducts varying according to self-reported MSDP. These adducts have been associated with environmental exposures and disease states including tobacco smoke, air pollution, and certain cancers [15,25,26]. The underlying chemistry and biology of these adducts as meaningful biomarkers of oxidant stress are also rapidly expanding [27]. To our knowledge, this is the first reported analysis to date on self-reported MSDP and cord blood HSA-Cys^34^ adductomics to explore the potential association between perinatal oxidant stress and prenatal tobacco exposure.

To date, the most reliable and well-characterized biomarker of tobacco smoke exposure remains serum cotinine [28], with large-scale epidemiologic studies demonstrating the correlation between self-reported MSDP behaviors and cord blood levels [29,30]. Despite their known sensitivity and clinical utility, toxin levels at birth do not reliably reflect the biological responses of the fetus and neonate—the most challenging to capture being perinatal oxidant stress. Thus, more robust approaches and meaningful biomarkers are needed to better understand how maternal smoking impacts the newborn infant, independent of the vast environmental exposures that follow postnatally during early infancy. While adductomics do not measure the actual toxin concentrations to which the fetus and newborn infant are exposed, they have, given their relative stability, advantages over absolute toxin levels in understanding complex, multifactorial mechanisms associated with maternal exposures and accompanying risk factors of preterm birth and low birth weight. While our analysis includes only a small sample representing current exposure, the cord blood profiles distinguishing current-smokers from former and never-smokers suggest that adductomics may be a sensitive approach to identify births impacted by oxidant-stress mediated pathology associated with acute smoking exposure surrounding birth. Although preliminary, our findings constitute the first study to date exploring these associations and provide avenues for future hypothesis-driven investigations.

The opportunity to conduct the reported secondary analysis was made possible by the large dataset of clinical data linked to targeted and untargeted adductomics. While the primary outcome upon which the original data were generated was neonatal BPD, an unanticipated finding was the possible association with MSDP. This association appeared to be influenced heavily by preterm birth, in which self-reported never-smoking was higher in full-term births (82%) versus preterm births (53%). Thus, it is likely that many of the adductomic differences reported in our secondary analysis were driven by gestational immaturity more so than by acute smoking exposure. An important discovery was that the adductomic profiles, when compared by MSDP report across the full gestational age spectrum, were distinct from those reported in our previous report comparing preterm and full-term births. Furthermore, matched and subgroup analyses by potential confounders, such as maternal hypertension and mode of delivery, yielded persistent distinction in the profiles of current-smokers versus the rest (Figure 5). A much larger sample, taking into account the complex interplay of covariates of maternal health and complications during pregnancy, is needed in order to identify meaningful biomarkers of MSDP exposure.

Several findings in this exploratory analysis demonstrate the potential utility of cord blood adductomic profiling to better understand the neonatal–perinatal exposome at birth. We found distinct profiles between current-smokers versus former and never-smokers, with 27 and 32 known adducts differentially increased in current-smokers versus the rest, respectively. Key adducts with the highest fold-changes included adducts implicated in known oxidant stress pathways: S-addition of S_2_O_3_H, S-addition of Cys, methylation, and S-addition of hCys. No significant differences in profiles were observed between former and never-smokers. However, these groups were relatively larger and likely more heterogeneous than the current-smoker group. Another intriguing hypothesis to explore in future studies is that the differences between groups reflect temporal dynamics related to smoking cessation, in which perinatal oxidant stress could be reversible or normalized to mimic never-smoker profiles after smoking cessation. Given the cross-sectional design and lack of confirmatory details on cessation timing within the former-smoker group, speculations about normalization or reversibility of oxidant stress warrant further investigation in studies with prospective, standardized collection of information on smoking onset and cessation during pregnancy.

Elevated oxidative stress is an important mediator of tobacco-induced pathology during pregnancy. Tobacco smoke contains thousands of chemicals, including reactive oxygen and nitrogen species (ROS/RNS) that damage DNA and proteins necessary for normal fetal development [25,26]. These electrophiles enter fetal circulation through the placenta, where they react with available proteins to form addition products, or “adducts” [13]. When bound to stable proteins such as HSA, these adducts become relatively more stable than scavenged reactive electrophiles [15] and thus may serve as reliable biomarkers of intrinsic and extrinsic environmental exposure [9]. HSA-Cys^34^ adducts potentially play a pivotal role in understanding human disease arising from exposures to electrophilic species [13,14]. HSA-Cys^34^ protein adductomic profiles from cord blood could serve as a molecular fingerprint of prenatal tobacco exposures in the weeks leading up to delivery. These profiles captured at birth in cord blood provide the first report applying adductomics to assess maternal smoking exposure in a pregnancy cohort.

The adducts identified in our study have been associated with pathological disease states in other populations. The predominance of adducts in small thiols, direct oxidation products, and reactive aldehyde groups reflects the major categories of oxidative stress biomarkers. Small thiols, known for their antioxidant capacity, may represent a response to high oxidative stress exposure. Direct oxidation products result from reactions between Cys^34^ and ROS, providing evidence of oxidative burden. Reactive aldehydes are associated with lipid peroxidation, a process that triggers cellular dysfunction in the setting of elevated oxidative stress. Several specific adducts warrant particular attention: S-methylthiolation.2 showed the highest fold change among known adducts (Log2FC = 2.65) in the current vs. never comparison, while the S-addition of hCys and S-addition of Cys methylation showed the largest fold changes in current vs. former-smokers. Additional thiol-conjugation adducts, including the Na adduct of S-CysGly and S-hCys, plus methylation, were also elevated, consistent with activation of multiple antioxidant and detoxification pathways. The identification of uniquely significant adducts in specific comparisons (A010 and A018 for current vs. never-smokers; A005, A006, A009, A019, A022, A026, and A032 for current vs. former-smokers) may reflect differing temporal dynamics of adduct formation and clearance.

Important limitations of this study include the relatively small number of current-smokers identified (N = 6), which limits generalizability. Adductomic analysis in a much larger cohort, along with more detailed information on smoking exposure, is the focus of our next set of investigations based upon the preliminary findings reported in this pilot analysis. Another limitation is that self-reported smoking status may be subject to misclassification due to social desirability bias during pregnancy [3]. Objective biochemical correlation (e.g., cotinine) of smoking status was not performed in this analysis. The cross-sectional design at delivery prevents assessment of temporal changes in adductomic profiles. Complete information on smoking frequency was not available, precluding dose-response analysis. Future studies with detailed information on timing of smoking cessation in the former-smoker group could address hypotheses regarding normalization and reversibility of oxidant stress as measured by the adductomic profiles. We did not assess functional consequences of the observed adductomic changes on pregnancy outcomes or infant health, although the impact on outcomes such as BPD should be investigated in a larger cohort of extremely preterm infants. Additionally, the study utilized archived samples from a single center, which provided uniformity but requires multi-center studies to assess generalizability of findings.

Lastly, the variability in analytic approaches across the emerging adductomics literature demonstrates that there is no universally accepted standardized analytic pipeline. Previously, we used traditional approaches coupled with data normalization to analyze adductomics data. In the present study, given the relatively smaller sample sizes and unbalanced groups, we opted to perform PERMANOVA on log10-transformed relative concentration values without additional normalization, using Euclidean distances; PERMDISP was performed on the same transformed data and distance matrix to directly assess whether within-group dispersion could confound the PERMANOVA result [31]. Smoking status was not a primary focus of the original study, but an unexpected association that prompted further exploration given the relevance to exposure science and the adductome. Thus, the results need to be interpreted with caution and validated in future prospective studies.

Future research should focus on several key areas. Longitudinal studies tracking adductomic changes throughout pregnancy in women who quit smoking could define the precise time course of adductomic recovery and identify the optimal timing for interventions. Investigation of dose-response relationships between smoking intensity, cotinine and adduct levels would strengthen causal inference and inform risk assessment. Studies linking specific adduct profiles to pregnancy outcomes and infant health would establish clinical relevance and identify which adducts best predict adverse outcomes. Detailed structural characterization of unknown adducts through advanced mass spectrometry techniques could reveal novel tobacco-specific biomarkers and identify previously unrecognized pathways of tobacco toxicity [19,32]. Extension to other environmental exposures such as e-cigarettes, secondhand smoke, and air pollution would broaden our understanding of environmental impacts on the prenatal exposome. The analytic approaches and pipelines developed here could be applied to other cohorts to characterize the full spectrum of prenatal environmental exposures and their biological consequences.

## 5. Conclusions

The application of adductomics to elucidate exposure pathways of MSDP is a novel and promising approach in pregnancy research. These exposure pathways could inform the development of targeted interventions and monitoring strategies during pregnancy. Linked to other high-resolution and high-throughput technologies, investigation of multiple omics could yield unprecedented findings and lead to innovative approaches in understanding and preventing environmental toxin-related pregnancy complications.

## Figures and Tables

**Table 1:** Maternal and infant characteristics by smoking exposure status.

	**Current Smoker** **N = 6**	**Former Smoker** **N = 26**	**Never Smoker** **N = 126**	*p* *
**Maternal Variables:**				
**Maternal age,** mean yrs ± SD	31.5 ± 6.4	33.0 ± 6.4	32.3 ± 5.2	0.79
**Maternal Race,** n (%)				0.06
*Black/African American*	4 (67%)	3 (11%)	25 (20%)	
*White*	1 (17%)	19 (73%)	65 (51%)	
*Other/Unknown*	1 (17%)	4 (15%)	36 (29%)	
**Maternal Ethnicity,** n (%)				0.70
*Hispanic or Latino*	0 (0)	5 (19%)	27 (21%)	
*Not Hispanic or Latino*	6 (100%)	21 (81%)	94 (75%)	
**Maternal pregnancy BMI,** mean ± SD	35.5 ± 5.7	31.8 ± 7.1	31.4 ± 6.2	0.29
**Preeclampsia**, n (%)	0 (0%)	3 (11%)	16 (13%)	1.00
**Chorioamnionitis**, n (%)	1 (17%)	4 (15%)	12 (9%)	0.39
**Mode of Delivery, n (%)**				0.02
*Vaginal*	2 (33%)	7 (27%)	69 (55%)	
*Cesarean*	4 (67%)	19 (73%)	57 (45%)	
**Rupture of Membranes,** n (%)				0.80
*Spontaneous*	2 (33%)	10 (38%)	55 (44%)	
*Artificial*	4 (67%)	16 (62%)	71 (56%)	
**Preterm Labor**, n (%)	4 (67%)	15 (57%)	62 (49%)	0.58
**Chronic Hypertension**, n (%)	3 (50%)	6 (23%)	7 (5%)	0.001
**Infant Outcomes:**				
**Gestational age,** mean wks ± SD	32.0 ± 6.0	29.2 ± 4.9	31.2 ± 6.1	0.28
**Birth weight,** mean gms ± SD	1918 ± 1136	1457 ± 983	1797 ± 1201	0.38
**Birth weight percentile,** mean ± SD	56.2 ± 26.1	65.3 ± 23.5	58.2 ± 25.4	0.40
**Male sex**, n (%)	5 (83%)	13 (50%)	56 (44%)	0.16
**Apgar (1-min),** median [IQR]	6.5 [6,9]	6 [4,8]	7 [4,8]	0.59
**Apgar (5-min),** median [IQR]	8.5 [8,9]	8 [7,9]	8 [6,9]	0.34
**NICU admission**, n (%)	5 (83%)	22 (85%)	85 (67%)	0.17

* *p*-values calculated using Analysis of Variance (ANOVA) or Kruskal–Wallis, where appropriate, for comparison of continuous data. Chi-square or Fisher’s exact tests were used to compare frequencies of categorical data among the 3 groups.

**Table 2:** PERMANOVA and PERMDISP results for cord blood adductomics by MSDP status.

**PERMANOVA**					
**Comparison**	**R^2^**	**% Variance**	**F**	**Raw *p***	**Adj. *p* (BH)**
Omnibus (3-group)	0.0267	2.7%	2.129	0.009	—
Former vs. Never	0.0043	0.4%	0.645	0.882	0.882
Current vs. Never	0.0258	2.6%	3.443	0.005	0.0075
Current vs. Former	0.1146	11.5%	3.884	0.003	0.0075
**PERMDISP (Homogeneity of Multivariate Dispersion)**					
**Comparison**	**F**	***p* value**	**Mean distance to centroid (Group 1)**		**Mean distance to centroid (Group 2)**
Omnibus (3-group)	2.000	0.149	—		—
Former vs. Never	2.061	0.152	5.735		5.310
Current vs. Never	2.312	0.138	5.735		4.882
Current vs. Former	0.474	0.522	5.310		4.882

## Data Availability

The original contributions presented in this study are included in the article/Supplementary Materials. Further inquiries can be directed to the corresponding author.

## Supplementary Materials

The following supporting information can be downloaded at: https://www.mdpi.com/article/10.3390/antiox15091078/s1, Data S1: Nomenclature and mass spectrometry parameters (m/z, added mass, retention times, PAR, and concentration) for all 105 known and unknown HSA-Cys^34^ adducts detected in cord blood plasma. Data S2: Complete annotated R analysis pipeline used to reproduce all statistical analyses and figures reported in the manuscript, including PCA, PERMANOVA, PERMDISP, and univariate volcano/violin plot screening. Data S3: (A) Pairwise statistical comparisons (group medians, log2 fold-change, 95% bootstrap confidence intervals, raw and adjusted *p*-values, and significance status) for all 56 known adducts across the three smoking-status comparisons. (B) Pairwise statistical comparisons (group medians, log2 fold-change, 95% bootstrap confidence intervals, raw and adjusted *p*-values, and significance status) for all 49 unknown adducts across the three smoking-status comparisons. Data S4: Maternal and infant clinical and demographic characteristics for all 158 study participants, linked to maternal smoking status and adductomics completion status. Data S5: Raw adduct concentrations (pmol/mg HSA) for all 158 cord blood plasma samples across all known and unknown adducts, with corresponding maternal smoking status. Data S6: Median adduct concentrations (pmol/mg HSA) for all known and unknown adducts, stratified by maternal smoking status.
