# Supplementary material for: Cord Blood Adductomic Profiling Provides Preliminary Insights on Perinatal Smoking Exposure: A Pilot Analysis
Source: Antioxidants (Basel). 2026 Aug 28;15(9):1078. doi: 10.3390/antiox15091078 (PMC13603371; doi:10.3390/antiox15091078)
Supplement: Supplementary file 1 [file antioxidants-15-01078-s001.zip › Data S6.pdf]

Known Adduct Concentrations in Median [IQR] According to Self-Reported Maternal Smoking.

Adduct	Annotation	Current_Smoker	Former_Smoker	Never_Smoker	P_Value	P_Adjusted
A034	S-Addition of Cys (-H2O)	5.99 [3.96-8.01]	1.60 [0.83-3.34]	1.69 [0.79-3.61]	0.02437	0.05054
A039	S-Addition of hCys (-H2O)	0.03 [0.01-0.08]	0.00 [0.00-0.01]	0.01 [0.00-0.03]	0.1202	0.1725
A040	S-Cys	848.87 [748.19-1027.31]	259.93 [146.76-404.15]	303.28 [186.00-563.14]	0.004424	0.02825
A044	S-Addition of hCys	77.83 [57.38-88.75]	12.96 [8.05-32.07]	21.19 [11.31-35.07]	0.009619	0.03529
A045	S-Addition of Cys, methylation	82.29 [58.10-105.09]	14.10 [10.00-33.63]	21.54 [11.46-39.45]	0.002493	0.02825
A047	Na adduct of S-Cys	66.43 [54.55-97.36]	16.10 [9.11-30.63]	20.70 [10.33-33.69]	0.00315	0.02825
A048	S-hCys, plus methylation (not Cys34)	2.46 [1.53-5.03]	0.79 [0.39-1.06]	0.66 [0.28-1.09]	0.003013	0.02825
A050	S-(N-acetyl)Cys	0.93 [0.82-2.00]	0.54 [0.32-0.89]	0.35 [0.18-0.79]	0.05164	0.09329
A051	S-Addition of CysGly	106.05 [84.97-156.69]	27.42 [12.77-53.56]	33.72 [15.87-72.14]	0.003832	0.02825
A052	S-CysGly, plus methylation (not Cys34)	2.38 [1.94-3.38]	1.11 [0.48-1.82]	1.01 [0.51-2.63]	0.05969	0.09832
A055	S-Addition of GluCys	1.37 [1.08-2.04]	0.35 [0.11-0.89]	0.35 [0.14-0.81]	0.0169	0.04508
A056	S-Addition of GSH	1.87 [1.64-2.08]	0.54 [0.34-1.01]	0.50 [0.28-1.05]	0.01218	0.03788
A033	S-Mercaptoacetamide	1.97 [1.26-2.68]	0.56 [0.30-1.10]	0.69 [0.31-1.30]	0.04105	0.07926
A035	S-Cys (possibly NH2 ‰ Õ OH, -H2O)	0.00 [0.00-0.00]	0.00 [0.00-0.00]	0.00 [0.00-0.01]	0.3871	0.4516
A041	S-Addition of Cys (NH2‰ ÕOH)	224.79 [183.29-292.04]	61.85 [38.44-114.36]	72.54 [47.33-135.31]	0.005782	0.02867
A046	S-Addition of hCys (NH2‰ ÕOH)	2.36 [1.12-2.75]	0.95 [0.64-2.15]	1.25 [0.68-2.80]	0.3804	0.4516
A049	S-Addition of CysGly (-H2O)	3.46 [3.02-6.15]	1.52 [0.86-3.38]	1.95 [1.03-3.20]	0.06345	0.1015
A053	Na adduct of S-CysGly	3.43 [2.04-5.60]	0.78 [0.38-1.39]	1.09 [0.55-2.03]	0.01549	0.04338
A054	K Adduct of S-CysGly	0.35 [0.09-0.62]	0.10 [0.06-0.14]	0.12 [0.04-0.26]	0.3807	0.4516

A019	S‰ÛÒ(O)‰ÛÒO‰ÛÒCH3	1.40 [1.16-1.61]	0.44 [0.19-0.55]	0.40 [0.20-1.04]	0.07238	0.1126
A026	Methylisocyanate adduct	3.10 [2.43-5.31]	1.29 [0.73-2.39]	1.71 [0.85-2.56]	0.04091	0.07926
A027	S-Addition of SO2	0.21 [0.09-0.37]	0.10 [0.05-0.18]	0.08 [0.03-0.14]	0.07675	0.1162
A029	S-Phenylation	0.28 [0.13-0.46]	0.08 [0.03-0.16]	0.08 [0.03-0.17]	0.2123	0.2824
A011	S-Cyanylation	4.05 [2.21-7.38]	1.28 [0.91-2.81]	1.73 [0.85-2.94]	0.05388	0.09428
A038	S-Addition of S2O3H	0.39 [0.35-0.47]	0.08 [0.03-0.21]	0.09 [0.02-0.19]	0.004541	0.02825
A043	Oxindole	0.77 [0.57-1.04]	0.17 [0.07-0.31]	0.27 [0.11-0.56]	0.01008	0.03529
A037	S-Methylethyl-sulfonylation	0.01 [0.00-0.02]	0.00 [0.00-0.01]	0.00 [0.00-0.01]	0.4272	0.4882
A008	Cys34-Gln cross-link (monooxidation), Cys34 Sulfinamide	43.72 [40.73-58.88]	22.19 [12.17-33.45]	24.42 [13.48-40.47]	0.0588	0.09832
A022	Cys34 sulfonic acid (trioxidation)	2.37 [1.70-3.17]	0.66 [0.22-1.16]	0.96 [0.30-2.39]	0.01992	0.04636
A014	Cys34 sulfinic acid (dioxidation)	2.58 [1.71-4.07]	0.90 [0.59-1.40]	1.08 [0.63-1.83]	0.0207	0.04636
A007	CH2 crosslink	2.29 [1.23-3.30]	1.30 [0.67-1.91]	1.17 [0.61-2.05]	0.2168	0.2824
A024	Na adduct of Cys34 sulfinic acid	0.80 [0.62-1.29]	0.23 [0.17-0.43]	0.27 [0.12-0.47]	0.007907	0.03406
A012	dehydrated form of Cys34 sulfinic acid plus methylation (not Cys34)	0.10 [0.01-262.75]	0.03 [0.01-0.12]	0.02 [0.00-0.09]	0.6622	0.7271
A013	dehydrated form of Cys34 sulfonic acid (trioxidation)	3.92 [2.97-4.33]	1.07 [0.68-1.78]	1.27 [0.77-2.30]	0.00207	0.02825
A020	Cys34 sulfinic acid plus methylation (not Cys34)	3.26 [2.04-3.28]	0.68 [0.21-1.01]	0.97 [0.32-1.93]	0.005401	0.02867
A025	Putative S-addition of acrolein	0.23 [0.07-0.69]	0.14 [0.09-0.21]	0.18 [0.08-0.38]	0.6876	0.7405

A028	S-Addition of crotonaldehyde	1.53 [1.39-1.65]	0.60 [0.28-1.04]	0.67 [0.27-1.06]	0.006144	0.02867
A030	S-Addition of tiglic aldehyde	0.25 [0.15-0.30]	0.09 [0.05-0.16]	0.10 [0.04-0.22]	0.2465	0.3137
A031	S-Addition of pyruvate or malonate semialdehyde	0.99 [0.72-1.30]	0.36 [0.15-0.58]	0.40 [0.20-0.72]	0.02402	0.05054
A036	S-addition of benzaldehyde or quinone methide	0.10 [0.06-0.29]	0.09 [0.05-0.18]	0.10 [0.04-0.28]	0.9791	0.9791
A001	-Lys from C-terminus	0.27 [0.13-0.38]	0.11 [0.04-0.36]	0.18 [0.04-0.54]	0.7086	0.7487
A003	Cys34‰ ÕDehydroalanine	0.00 [0.00-0.25]	0.02 [0.00-0.06]	0.02 [0.00-0.07]	0.765	0.7933
A004	Cys34‰ ÕOxoalanine or formylglycine	0.24 [0.17-0.43]	0.14 [0.05-0.25]	0.10 [0.04-0.27]	0.1473	0.2062
A002	Cys34‰ ÕGly	1.21 [0.78-1.41]	0.25 [0.11-0.53]	0.39 [0.21-0.78]	0.004411	0.02825
A009	Methylation (not at Cys34)	35.52 [26.86-40.88]	15.82 [9.97-27.48]	20.07 [10.85-30.57]	0.04672	0.08721
A010	S-Sodiation	46.82 [43.39-59.78]	23.84 [12.72-37.71]	22.88 [11.90-38.84]	0.01091	0.03595
A016	Ethylene oxide adduct	0.31 [0.28-0.35]	0.21 [0.11-0.42]	0.21 [0.10-0.39]	0.4458	0.4993
A023	Acrylonitrile adduct	10.04 [7.90-13.22]	3.88 [1.91-6.17]	3.32 [1.40-6.90]	0.008816	0.03526
A032	S-Addition of mercaptoacetic acid	1.70 [0.84-2.68]	0.34 [0.22-0.97]	0.69 [0.33-1.44]	0.01781	0.04534
A042	S-Addition of BDE	0.01 [0.00-0.05]	0.02 [0.01-0.06]	0.01 [0.00-0.05]	0.7829	0.7972
A005	T3 dimer	1374.74 [1280.97-1986.89]	602.62 [331.77-977.25]	709.27 [397.79-1136.73]	0.0141	0.04154
A015	K adduct of T3	3.11 [2.69-3.17]	0.65 [0.43-1.39]	0.94 [0.45-1.85]	0.002107	0.02825
A017	S-Methylthiolation.1	0.59 [0.34-0.87]	0.24 [0.11-0.37]	0.24 [0.12-0.59]	0.151	0.2062
A018	S-Methylthiolation.2	0.27 [0.13-0.31]	0.04 [0.02-0.11]	0.04 [0.01-0.12]	0.09036	0.1332
A021	S-Methylthiolation	0.17 [0.10-0.27]	0.04 [0.02-0.14]	0.05 [0.02-0.22]	0.2967	0.3692
A006	Unmodified T3	1103.20 [1071.17-1493.30]	503.51 [304.24-933.27]	621.42 [340.84-1034.53]	0.0191	0.04636

Median adduct concentrations and interquartile values are in pmol adduct/mg HAS, multiplied by 10^3. P-values determined by Kruskal-Wallis, with adjusted P-value determined by Benjamini-Hochberg FDR.

Unknown Adduct Concentrations in Median [IQR] According to Self-Reported Maternal Smoking.

Adducts	Current_Smoker	Former_Smoker	Never_Smoker	P_Value	P_Adjusted
Unknown (-10.07 Da)	8.37 [5.21-9.72]	1.74 [0.76-5.19]	3.42 [1.37-6.62]	0.06753	0.2364
Unknown (192 Da)	1.56 [1.25-3.05]	0.55 [0.32-1.09]	0.53 [0.26-1.32]	0.01876	0.1149
Unknown (8.85 Da)	0.00 [0.00-0.05]	0.01 [0.00-0.03]	0.01 [0.00-0.04]	0.9121	0.9164
Unknown (34.92 Da)	0.05 [0.01-0.07]	0.01 [0.00-0.01]	0.01 [0.00-0.02]	0.1751	0.351
Unknown (53.11 Da)	6.88 [6.64-10.39]	3.73 [2.19-5.66]	4.01 [2.14-6.59]	0.04277	0.1746
Unknown (likely S-addition of an aldehyde)	2.12 [1.28-2.92]	0.98 [0.34-1.69]	1.04 [0.55-1.80]	0.08888	0.2722
Unknown (62.01 Da)	0.01 [0.00-0.03]	0.00 [0.00-0.02]	0.00 [0.00-0.02]	0.699	0.7799
Unknown (101.06 Da)	0.47 [0.33-0.57]	0.05 [0.02-0.13]	0.06 [0.02-0.14]	0.002896	0.07096
Unknown (109.03 Da)	0.01 [0.00-0.02]	0.02 [0.00-0.04]	0.01 [0.00-0.06]	0.8966	0.9164
Unknown (111.03 Da)	0.03 [0.02-0.04]	0.00 [0.00-0.03]	0.00 [0.00-0.01]	0.03446	0.1535
Unknown (126.08 Da)	0.01 [0.00-0.01]	0.01 [0.00-0.04]	0.01 [0.00-0.03]	0.6989	0.7799
Unknown (137.06 Da)	0.29 [0.23-2.06]	0.25 [0.09-0.48]	0.36 [0.16-0.74]	0.1934	0.351
Unknown (138.06 Da)	0.24 [0.13-0.52]	0.14 [0.04-0.38]	0.19 [0.05-0.42]	0.7162	0.7799
Unknown (143 Da)	0.94 [0.72-1.09]	0.27 [0.17-0.70]	0.44 [0.20-0.76]	0.04762	0.1795
Unknown (151.99 Da)	2.19 [1.24-3.69]	1.05 [0.47-1.85]	1.07 [0.59-1.87]	0.3159	0.4868
Unknown (153.05 Da)	0.84 [0.57-1.68]	0.50 [0.34-1.05]	0.53 [0.23-1.09]	0.2309	0.3902
Unknown (156.09 Da)	0.00 [0.00-0.00]	0.00 [0.00-0.01]	0.00 [0.00-0.03]	0.7603	0.8098
Unknown (156.96 Da)	4.34 [2.86-5.16]	0.88 [0.49-1.73]	1.00 [0.51-2.11]	0.008615	0.08443
Unknown (180.02 Da)	3.21 [2.73-4.44]	0.87 [0.31-2.25]	1.11 [0.48-2.23]	0.007689	0.08443
Unknown (183.02 Da)	0.11 [0.03-0.19]	0.03 [0.01-0.10]	0.06 [0.03-0.19]	0.1688	0.351
Unknown (185.02 Da)	0.00 [0.00-0.02]	0.01 [0.00-0.03]	0.01 [0.00-0.04]	0.9164	0.9164
Unknown (202.02 Da)	0.00 [0.00-0.00]	0.00 [0.00-0.00]	0.00 [0.00-0.00]	0.2992	0.4868
Unknown (212.32 Da)	0.03 [0.01-0.04]	0.01 [0.00-0.02]	0.01 [0.00-0.03]	0.328	0.4868
Unknown (247.1 Da)	1.57 [0.80-2.15]	0.38 [0.13-0.84]	0.49 [0.19-0.82]	0.01457	0.102
Unknown (262.1 Da)	0.00 [0.00-0.00]	0.01 [0.00-0.02]	0.01 [0.00-0.03]	0.1636	0.351
Unknown (319.09 Da)	7.53 [6.43-10.25]	2.95 [2.18-6.74]	4.35 [2.19-8.64]	0.1751	0.351
Unknown (346.14 Da)	0.08 [0.05-0.09]	0.06 [0.05-0.11]	0.09 [0.03-0.19]	0.7097	0.7799
Unknown (351.07 Da)	1.07 [0.91-1.53]	0.15 [0.03-0.40]	0.15 [0.02-0.52]	0.0272	0.1463
Unknown (360.19 Da)	0.28 [0.15-0.51]	0.11 [0.05-0.41]	0.24 [0.08-0.51]	0.1888	0.351

Unknown (388.2 Da)	3.01 [1.12-4.89]	0.12 [0.04-0.25]	0.20 [0.05-0.76]	0.002038	0.07096
Unknown (461.2 Da)	3.12 [1.70-3.38]	1.11 [0.62-2.16]	1.31 [0.72-2.26]	0.177	0.351
Unknown (+476 Da)	0.01 [0.01-0.09]	0.08 [0.03-0.17]	0.05 [0.01-0.13]	0.1652	0.351
Unknown (+489 Da)	0.54 [0.38-0.95]	0.34 [0.14-0.53]	0.32 [0.15-0.65]	0.2087	0.3653
Unknown (+495.21 Da, M49,OS39)	1.65 [0.86-2.22]	0.68 [0.43-1.31]	0.86 [0.47-1.52]	0.4442	0.6219
Unknown (+509.21 Da)	0.01 [0.01-0.17]	0.01 [0.00-0.03]	0.02 [0.00-0.05]	0.5869	0.7189
Unknown (-12.96 Da)	0.06 [0.03-0.09]	0.01 [0.01-0.05]	0.02 [0.01-0.06]	0.3378	0.4868
Unknown (+154.35 Da)	0.07 [0.05-0.10]	0.01 [0.00-0.02]	0.01 [0.00-0.04]	0.02986	0.1463
Unknown (+155.07 Da)	7.56 [4.79-10.78]	1.58 [0.84-3.29]	2.32 [1.10-3.94]	0.007622	0.08443
Unknown (+320.97 Da)	0.02 [0.01-0.07]	0.01 [0.00-0.02]	0.01 [0.00-0.03]	0.6589	0.7799
Unknown (322.08 Da)	0.03 [0.01-0.09]	0.01 [0.00-0.02]	0.01 [0.00-0.02]	0.1103	0.3002
Unknown (+340.08 Da)	0.01 [0.00-0.03]	0.00 [0.00-0.01]	0.00 [0.00-0.01]	0.4951	0.6557
Unknown (+545.22 Da)	1.36 [0.92-1.66]	0.39 [0.20-0.90]	0.54 [0.24-1.00]	0.0753	0.246
Unknown (-12.96 Da), 2nd	0.28 [0.18-0.61]	0.08 [0.04-0.33]	0.11 [0.05-0.32]	0.188	0.351
Unknown (+154.35 Da), 2nd	0.04 [0.02-0.07]	0.01 [0.00-0.02]	0.01 [0.00-0.03]	0.1027	0.2962
Unknown (+155.07 Da), 2nd	3.57 [2.62-4.05]	0.89 [0.48-1.64]	1.41 [0.64-2.45]	0.01363	0.102
Unknown (+320.97 Da), 2nd	5.12 [2.72-8.48]	3.06 [2.13-6.82]	3.74 [2.18-7.71]	0.5656	0.7107
Unknown (+322.08 Da), 2nd	0.07 [0.01-0.15]	0.01 [0.00-0.06]	0.01 [0.00-0.04]	0.4929	0.6557
Unknown (+340.08 Da), 2nd	0.00 [0.00-0.04]	0.00 [0.00-0.00]	0.00 [0.00-0.01]	0.5489	0.7078
Unknown (+545.22 Da), 2nd	0.96 [0.64-1.39]	0.42 [0.20-1.29]	0.55 [0.27-1.05]	0.3313	0.4868

Median adduct concentrations and interquartile values are in pmol adduct/mg HSA, multiplied by 10^3. P-values determined by Kruskal-Wallis, with adjusted P-value determined by Benjamini-Hochberg FDR.
